# A Goal Programming Model for Nurse Shift Scheduling Incorporating Flexible Constraints: A Case Study in an Operating Room Department

**DOI:** 10.3390/healthcare14182955

**Published:** 2026-09-10

**Authors:** Mert Demircioğlu, Hazal Ezgi Mutlu

**Affiliations:** 1 Department of Business Administration, Çukurova University, Adana 01330, Türkiye; 2 Department of Management Information Systems, Çağ University, Mersin 33800, Türkiye; hazalezgiozbek@cag.edu.tr

**Keywords:** nurse scheduling, shift scheduling, goal programming, flexible constraints, operating room, optimization, healthcare management

## Abstract

**Highlights:**

**What are the main findings?**

- A goal programming model incorporating five flexible constraints informed by nurses’ preferences and institutional requirements limited weekly night-shift assignments to a maximum of two per nurse and eliminated direct night-to-day shift transitions in a 22-room operating room department.
- Standardizing monthly working days to an 18–20-day range from an irregular 14–26-day range (an 86% reduction in standard deviation, from 3.51 to 0.49) and eliminating all identified rest-period violations for 16 h and 24 h subcontracted duties demonstrated that systematic optimization can improve compliance with predefined scheduling objectives relative to manual scheduling.

**What are the implications of the main findings?**

- Integrating nurse preferences as formal goal constraints within a goal programming model provides nurse managers with a decision-support tool that improves the equality of scheduled working days and may support reduced workload-related fatigue in surgical settings.
- The model’s constraint structure and Python-based implementation can be adapted to other hospital departments and healthcare institutions by adjusting staffing parameters and penalty weights, supporting broader adoption of systematic, preference-informed nurse scheduling practices, subject to validation in other settings.

**Abstract:**

**Background/Objectives:** Operating room nurse scheduling is a complex healthcare optimization problem. Operating room settings are particularly challenging because permanent and subcontracted nurses operate under complex 16 h and 24 h shift structures, with continuous surgical coverage requirements and recovery-period requirements. To our knowledge, few existing models simultaneously integrate nurse preferences, recovery-period requirements, and heterogeneous shift structures within a unified goal programming framework. This study aims to develop and implement a goal programming model that incorporates nurses’ needs and preferences as flexible constraints to optimize shift scheduling in an operating room department. **Methods:** This single-center case study combined a qualitative component, an analysis of scheduling records, and mathematical optimization modeling. It was conducted at the operating room department of a public hospital in Türkiye employing 37 permanent and 7 subcontracted nurses in the shift rotation, together with a head nurse responsible for the roster. The hospital was selected purposively as a high-volume public center with a dual-tier staffing model and a fully manual scheduling process; semi-structured interviews were then conducted with all 37 permanent nurses and the head nurse (*n* = 38). The seven subcontracted nurses were not interviewed; the constraints applying to them were derived from national regulatory guidance and operational information provided by the head nurse. Scheduling requirements were formalized as five flexible constraints informed by nurses’ preferences and institutional requirements and incorporated into a goal programming model alongside obligatory coverage and staffing constraints. Penalty weights were calibrated through structured consultation with the head nurse. The model was solved to proven optimality using Python with the OR-Tools CP-SAT solver. **Results:** The optimized 28-day schedule eliminated direct night-to-day shift transitions, which, under the manual schedule, affected approximately three nurse assignments per week. Weekly night shifts were limited to a maximum of two per nurse in every planning block, a limit that individual nurses exceeded under the manual system. Monthly working days were standardized to a range of 18–20 days (mean = 19.84, SD = 0.49) from an irregular 14–26-day range (mean = 20.57, SD = 3.51), an 86% reduction in the standard deviation. All identified rest-period violations for subcontracted nurses on 16 h and 24 h duties were eliminated in the optimized schedule. **Conclusions:** A goal programming model integrating flexible constraints informed by nurses’ preferences and institutional requirements generated a schedule with greater equality in the distribution of monthly working days and improved compliance with the predefined scheduling objectives compared with the historical manual schedule. The model offers nurse managers a potentially adaptable decision-support tool that requires prospective validation in other settings. Future work should extend the model to incorporate dynamic patient demand, cost optimization, and multi-department scheduling scenarios.

## 1. Introduction

Operating room nurse scheduling is a complex healthcare optimization problem due to heterogeneous shift structures, emergency surgical demand, and strict rest-period requirements. Unlike general ward scheduling, operating room environments must reconcile continuous surgical coverage, unpredictable emergency procedures, and recovery-period requirements following extended 16 h and 24 h duties while simultaneously accommodating individual nurse preferences. Inefficient scheduling in this context adversely affects job performance and increases the risk of clinical error [1,2]. Demanding working conditions burden nurses with excessive assignments and inadequate rest, leading to dissatisfaction and reduced performance [1,2,3]; qualitative work with staff nurses and nurse managers identifies the schedule itself as a central determinant of job satisfaction and of the intention to remain in post [4]. Public health crises such as the COVID-19 pandemic compound these challenges further, raising operational complexity [5].

These pressures take a distinctive form in the operating room. Surgical suites must maintain continuous coverage across elective lists, delayed cases, and unscheduled emergencies, so staffing cannot follow a fixed ward roster [6]. Coverage beyond the standard 08:00–16:00 working day is typically sustained through extended 16 h and 24 h duties, each carrying its own mandatory recovery period, and, as in the department examined here, through a second tier of subcontracted staff whose contractual conditions differ from those of permanent nurses. The resulting roster must reconcile three interacting requirements simultaneously: uninterrupted surgical coverage, differentiated recovery entitlements attached to heterogeneous duty lengths, and an equitable distribution of night work and monthly workload across a staff group with individual preferences; comparable multi-requirement rostering problems arise in other acute settings, such as the emergency department [7]. Where these requirements are reconciled manually, night shifts accumulate unevenly, nurses are assigned to day shifts immediately after night duty, and monthly working days diverge widely between individuals. Shift-work research associates these patterns with impaired recovery and elevated fatigue risk [8,9]. This configuration motivates the methodological choice set out below.

A wide range of optimization approaches has been applied to nurse scheduling, which can be grouped by methodological category; recent reviews map the field in full [10]. Metaheuristic methods, including tabu search [11,12] and memetic algorithms [13], offer computational efficiency for large-scale instances but cannot guarantee global optimality and often require extensive parameter tuning. Integer and mixed-integer programming (MILP) formulations, applied to scheduling problems generally [14,15] and to healthcare staff rostering specifically [16,17,18,19,20,21], provide provably optimal solutions and have been widely applied, including by Trilling et al. [16], who scheduled anesthesia nurses across a nine-theatre surgical suite, a setting closely analogous to the present study; by Mystakidis et al. [22] in an oncology rostering case study; and by Saemi [23], who extends the formulation to a reserve nursing pool solved through a hybrid metaheuristic (a dual-tier staffing arrangement comparable in structure to the permanent–subcontracted division examined here); however, these formulations typically absorb preference satisfaction into penalty terms within a single objective rather than representing each requirement as an explicit goal constraint with its own deviation variables and priority weight. Goal programming has been applied to nurse scheduling by Mohammadian et al. [24], Ariyani et al. [25], Agyei et al. [26], and Torres-Ramos et al. [27], while Burke et al. [13] incorporated nurse preferences and contractual conditions, Bagheri et al. [28] applied stochastic programming to the problem, and Wong et al. [29] and Jafri et al. [30] developed soft-constraint models. Beyond nursing, Chaker et al. [31] developed a goal programming model solved via a GRASP metaheuristic to minimize workload, travel time, premium costs and radiation exposure in multi-skill personnel scheduling, illustrating how weighted deviation objectives accommodate competing operational and well-being criteria. Preference satisfaction has also been pursued through hybrid exact and heuristic schemes: Mohd Razali et al. [32] combined integer linear programming with a genetic algorithm to maximize nurses’ shift and off-day preferences, reporting substantial gains in preference fulfillment relative to manual schedules. Aydas et al. [33] addressed short-term schedule adjustment under dynamic patient demand, and Narlı and Derse [34] optimized crew scheduling in an intensive care unit. Within surgical settings specifically, Gür et al. [35] combined constraint programming with goal programming to assign surgical teams and schedule operations, and Al Amin et al. [6] reviewed two decades of operating room scheduling research. Constraint-programming solvers have also gained ground in this domain: Jiang et al. [36] reported that a CP-SAT formulation of the nurse scheduling problem attained feasible, constraint-satisfying rosters substantially faster than a genetic-algorithm baseline, supporting the use of an exact constraint-programming solver in the present study.

Despite this breadth of research, an important gap remains: many previous studies focus either on operational efficiency or on individual staff preferences, and, to our knowledge, few simultaneously integrate human, operational, and regulatory requirements in operating room environments characterized by 16 h and 24 h shift structures. Limited evidence is available on the explicit treatment of night-to-day shift transitions, a pattern associated with accelerated fatigue and reduced care quality [8,37], as a formal scheduling goal in prior goal programming studies.

Goal programming suits this context. It handles multi-objective problems involving flexible constraints and human preferences, allowing the balancing of potentially conflicting objectives such as operational coverage, workload equity, and nurse well-being. Unlike single-objective MILP, goal programming treats nurse preferences as formal goal constraints rather than post hoc adjustments; furthermore, unlike metaheuristics, goal programming solved with an exact solver guarantees global optimality. This combination of multi-objective flexibility and solution optimality makes goal programming a strong methodological choice for operating room scheduling.

To address the identified gap, the present study develops and implements a goal programming model that incorporates nurses’ preferences and institutional requirements as flexible constraints for shift scheduling in an operating room department. The specific objectives are (1) to identify scheduling inefficiencies in the current manual system through semi-structured interviews with nurses and the head nurse; (2) to construct a goal programming model with five flexible constraints reflecting nurses’ needs and institutional requirements; (3) to solve the model using an exact constraint-programming solver and evaluate the optimized schedule against the current system; and (4) to assess the extent to which the optimized schedule improves compliance with the predefined scheduling objectives relative to the historical schedule.

The scientific contribution of this study lies not in the number of flexible constraints employed, but in their simultaneous integration within a single optimization framework. Specifically, the proposed model concurrently integrates heterogeneous 16 h and 24 h shift structures, minimum recovery-period requirements, and preferences elicited from nurses within the specific context of operating room scheduling, a combination that, to our knowledge, has received limited attention in prior goal programming research. Penalty weights for the flexible constraints were calibrated through structured consultation with the head nurse, ensuring practical relevance to the institutional context.

To position the present study within the existing goal programming literature on nurse scheduling, Table 1 summarizes how prior models address the key dimensions relevant to operating room scheduling. While nurse preferences and soft constraints are well established in this body of work, most studies focus on general ward settings and do not jointly account for the operational realities of surgical environments. In particular, the simultaneous treatment of heterogeneous 16 h and 24 h shift structures, mandatory recovery periods, and a dual-tier permanent–subcontracted staffing model has received limited attention in the goal programming literature identified in our review. As Table 1 shows, what sets the present study apart is the integration of all these dimensions within a single optimization framework tailored to the operating room context.

## 2. Materials and Methods

Nurses in the study department work within a two-shift daily structure: a day shift from 08:00 to 16:00 and a night shift from 16:00 to 24:00, both covered by permanent nurses. In addition, subcontracted nurses cover extended duties: a 16 h duty running from 16:00 on one day to 08:00 the following day, and a 24 h duty running from 08:00 on one day to 08:00 the following day. The two-shift structure follows national shift-work guidance for healthcare institutions [39]. The department additionally applies a recovery period of two days following a 16 h duty and three days following a 24 h duty; the regulatory basis for post-duty rest, and the discretion the institution exercises in setting these periods, are set out in Section 2.3.4. The physiological rationale for recovery windows of this kind after extended night duty is documented in the shift-work literature [9,40]. Table 2 summarizes the shift types, their working hours and duration, the eligible staff category, and the corresponding minimum recovery period.

### 2.1. Study Design, Setting and Staffing Structure

This study is a single-center case study combining three components: a qualitative component based on semi-structured interviews with nursing staff, an analysis of historical scheduling records, and mathematical optimization modeling. The qualitative component identified recurring scheduling concerns and translated them into goal constraints; the scheduling records component provided the baseline against which the optimized schedule was compared; and the modeling component generated the optimized schedule.

This study was conducted in the operating room department of a public hospital in Türkiye. The department comprises 22 operating rooms and employs 44 nursing staff who take part in the shift rotation: 37 permanent nurses employed directly by the hospital (32 females, 5 males) and 7 subcontracted nurses (all females) assigned exclusively to the extended 16 h and 24 h duties. The department is additionally managed by a head nurse, who is responsible for preparing the roster but does not take surgical or night duties and is therefore not among the 44 rotating staff; she was interviewed in a managerial capacity, so that the interview sample comprises 38 participants in total. The department performs an average of 150–180 surgical procedures per week. Under the standard staffing protocol, two nurses are assigned per operating room; however, due to chronic staffing shortages, three nurses are currently responsible for two rooms in practice. Subcontracted nurses supplement coverage for procedures scheduled after 16:00, as permanent nurses’ standard working hours are between 08:00 and 16:00.

Participant selection followed a two-stage purposive sampling strategy. In the first stage, the study site was selected purposively: a public hospital with a high-volume operating room department (22 operating rooms; 150–180 procedures per week), a dual-tier permanent–subcontracted staffing model, and a fully manual scheduling process. In the second stage, all 37 permanent nurses working in the department and the head nurse were invited to participate (*n* = 38 invited). The inclusion criteria were current employment as a nurse in the operating room department and participation in the departmental shift rotation; all invited participants consented and took part.

The seven subcontracted nurses were not included in the interview process. They are employed through an external staffing contract, and their working conditions, including the mandatory recovery periods following 16 h and 24 h duties, are governed by the terms of the service contract and by the national regulatory framework set out in Section 2.3.4, rather than by individual preference. The constraints applying to them were therefore derived from regulatory requirements and from operational information provided by the head nurse and not from the preferences of the subcontracted nurses themselves. This is acknowledged as a limitation of the preference-elicitation component of this study.

### 2.2. Data Collection

Data collection was conducted in two phases, between December 2025 and January 2026. In the first phase, scheduling records covering the four-week period from 1 to 28 December 2025 were retrieved from the hospital’s human resources system, containing detailed information on each nurse’s shift assignments. This period was selected as representative of routine departmental operation because it contained no public holidays, no departmental reorganization or change in staffing establishment, and a surgical case volume consistent with the departmental weekly average.

In the second phase, a single researcher conducted semi-structured individual interviews with all 37 permanent nurses and the head nurse (*n* = 38), between 12 and 23 January 2026; each interview lasted approximately 15–30 min. Participant characteristics are reported in Table 3. Prior to data collection, verbal institutional permission was obtained from the hospital administration. This study involved only minimal-risk data (anonymized work-hour records, shift patterns, scheduling preferences, and staff characteristics compiled from departmental staffing records, with no personal health information, patient data, or sensitive identifiers), and on that basis, the manuscript was initially submitted before formal ethics committee review had been completed. Review was applied for on 21 April 2026 and completed thereafter: the Institutional Review Board of Çukurova University (Scientific Research and Publication Ethics Committee in the Field of Social and Humanities Sciences) approved the study on 30 April 2026 (Decision No. 15) and, in a supplementary decision issued on 6 July 2026 (Decision No. 5), explicitly confirmed that the study was conducted retrospectively and complies with ethical principles. All participating nurses provided written informed consent prior to participation, using the form reproduced in Appendix A, and all data were anonymized prior to analysis.

The characteristics of the nursing staff were compiled from departmental staffing records and provided in anonymized form by the head nurse. Of the 37 permanent nurses, 32 (86.5%) were female and 5 (13.5%) male. They had a mean age of 37.32 years (SD = 6.19; median 36, range 24–52) and a mean of 15.27 years of nursing experience (SD = 6.39), of which a mean of 13.57 years (SD = 6.07) had been spent in operating room nursing. The head nurse was 40 years old, with 18 years of nursing experience, of which 15 were in the operating room. Full participant characteristics are given in Table 3.

The interview questions were developed through a two-source process combining prior literature and field input. First, an initial set of questions was derived from recurring themes in the nurse scheduling literature, specifically night-shift frequency and accumulation [27,29], rest-period and recovery adequacy [1,2], workload equity and consecutive-shift patterns [19,41], and individual preference satisfaction [24,38]. Second, these questions were reviewed and refined with the head nurse to ensure relevance to the specific operational conditions of the operating room department, including its 16 h and 24 h shift structures and dual-tier staffing model. This combined literature- and field-grounded approach ensured that the questions captured both established scheduling concerns and context-specific factors. The interviews accordingly focused on scheduling preferences, perceived workload burdens, rest-period adequacy, and requests regarding night-shift frequency and consecutive-shift patterns.

Responses were recorded as written notes during each interview. Interview notes were analyzed thematically by a single researcher following an inductive coding process: notes were read repeatedly, recurring scheduling concerns were coded, and codes expressed by multiple participants were grouped into themes. Three recurring themes were identified in the interviews: (1) excessive accumulation of night shifts within a single week; (2) insufficient recovery time between a night shift and a subsequent day assignment; and (3) perceived inequity in the distribution of monthly working days. A fourth requirement, adequate rest following extended 16 h and 24 h duties, was not raised in the interviews, since those duties are worked only by the subcontracted nurses, who were not interviewed; this requirement was derived from national regulatory guidance and from operational information provided by the head nurse. Each theme was translated into a corresponding goal constraint in the mathematical model, together with the regulatory recovery requirements applying to subcontracted staff; this mapping is presented in Table 4. The analysis was interpretive rather than quantitative: the number of participants raising each theme was not tallied, so the themes are reported as recurring concerns rather than as counted proportions. The use of a single coder without independent verification is acknowledged as a methodological limitation. Penalty weights for each goal constraint were determined collaboratively with the head nurse.

### 2.3. Mathematical Model

Shift scheduling was modeled as a goal programming problem. The hospital employs two categories of nurses. The first group is the permanent hospital staff, who work during the day and sometimes at night and are represented in the model by the variable “X”. The second group comprises the subcontracted nurses designated for the 16 h and 24 h shifts, represented by the “Y” and “Z” variables.

The constraints are established in two distinct ways, categorized as obligatory and flexible constraints. The obligatory constraints define the feasible region: they ensure that the required number of nurses is present on each day and shift, and they enforce the structural conditions governing the subcontracted staffing pool. The five flexible constraints correspond to the scheduling concerns identified in the interviews and to the regulatory recovery requirements. The first addresses the accumulation of night duties, which, under manual scheduling, could exceed two nights within a single week for an individual nurse. The second prevents a nurse assigned to a night shift from being assigned to the day shift on the following day, a transition that occurred routinely under the manual system. The third establishes a minimum and maximum number of working days per four-week cycle, so that monthly workload, and with it the incidence of overtime, is distributed more evenly across nurses. The fourth and fifth enforce the recovery periods that follow 16 h and 24 h duties for subcontracted nurses. Each of these five requirements is represented as a goal constraint with deviation variables, and deviations are penalized in the objective function according to the weight assigned to that requirement.

An exact constraint-programming solver was used to solve the model. The penalty weights attached to the goal constraints express the relative priority of each requirement, so that the solver resolves competing objectives in the order the department considers most important. The model is set out below.

#### 2.3.1. Indexes and Parameters

Four indexes are used in this model:

i: nurse’s index (i = 1,2, …, 37),

j: day’s index (j = 1, …, 28),

k: shift’s index (1: day, 2: night),

l: subcontracted nurse’s index (l = 1, …, 7),

$R_{jk}$: required number of nurses on day j, shift k.

The model uses the following input parameters, fixed prior to optimization: the planning horizon (28 days, organized into four 7-day blocks); the number of permanent nurses (37) and subcontracted nurses (7); the shift structure (day and night shifts, plus 16 h and 24 h subcontracted duties, as set out in Table 2); the daily staffing requirement R_jk_; the monthly working-day bounds (18 and 20 days; the departmental target of 19 days is not itself a model parameter); the weekly night-shift target (2); the recovery-period lengths (2 days following a 16 h duty and 3 days following a 24 h duty); and the penalty weights C_n_^−^ and C_n_^+^ associated with each goal constraint. Of these, R_jk_ is the only parameter that varies by day and shift. The values used follow the department’s typical operating pattern and are the average requirements recorded in December 2025: 24 nurses on a weekday day shift, 14 on a weekend day shift, and 8 on any night shift. Within the model, these figures are exact: Equations (1) and (2) require the corresponding number of nurses to be on duty on every day of the horizon. Each figure is nevertheless a mean, and the requirement on an individual day runs above or below it with surgical load, so the model is solved against a representative demand profile rather than a day-specific one.

#### 2.3.2. Decision Variables

In the model, variable X consists of normal daily shifts. Y and Z represent the night duties of the 7 subcontracted nurses (indexed by l), where Y denotes 16 h duties and Z denotes 24 h duties. All three are binary. For each goal constraint 1 to 7, a pair of non-negative integer deviation variables measures the departure from the target value. Each goal constraint is written in the form (achieved value) − S_n_^−^ + S_n_^+^ = target; accordingly, S_n_^−^ denotes the amount by which the achieved value exceeds the target, and S_n_^+^ is the amount by which it falls below the target.

$$\begin{array}{cc} X_{ijk}= & \left\{\begin{array}{c} 1,\;if\;the\;ith.\;nurse\;is\;working\;on\;jth.\;day\;on\;kth.\;shift\\ \;0,\;in\;other\;cases \end{array}\right.\\ Y_{ljk}= & \left\{\begin{array}{c} 1,\;if\;the\;lth.\;nurse\;has\;16\;h\;night\;duty\;on\;jth.\;day\;on\;kth.\;shift\\ \;0,\;in\;other\;cases \end{array}\right.\\ Z_{ljk}= & \left\{\begin{array}{c} 1,\;if\;the\;lth.\;nurse\;has\;24\;h\;night\;duty\;on\;jth.\;day\;on\;kth.\;shift\\ \;0,\;in\;other\;cases \end{array}\right. \end{array}$$

$S_{n}^{-},\;S_{n}^{+}$: negative and positive deviation variables for goal constraint n (n = 1, …, 7).

In addition, S_8lj_, S_9lj_ ∈ {0, 1} are binary violation indicators for the 16 h and 24 h recovery-period requirements respectively, for subcontracted nurse l on day j; each takes the value 1 when the corresponding recovery period is not respected.

#### 2.3.3. Obligatory Constraints

The obligatory constraints are set out below.

1. For every day and night shift, the number of working nurses needs to be met.

For the day shift:

(1) $$\sum_{i=1}^{37}X_{ij1}+\;\sum_{l=1}^{7}Z_{lj1}\geq \;R_{j1};\mathrm{all}\;j:\;1,\ldots ,28$$

For the night shift:

(2) $$\sum_{i=1}^{37}X_{ij2}+\sum_{l=1}^{7}Z_{lj2}+\sum_{l=1}^{7}Y_{lj2}\geq \;R_{j2};\mathrm{all}\;j:\;1,\ldots ,28$$

A 24 h duty spans both the day and the night period of the calendar day on which it begins (Table 2); the corresponding variable Z_lj1_ therefore contributes to day-shift coverage in Equation (1), while Z_lj2_ contributes to night-shift coverage in Equation (2), the two being tied together by Equation (6). A 16 h duty begins at 16:00 and therefore contributes nothing to day-shift coverage; Equation (3) fixes the day-shift component of the 16 h duty variable to zero accordingly. Stand-alone day shifts are represented only by X and are therefore reserved for permanent nurses by construction.

Coverage is expressed as an inequality rather than an equality because the department operates under a chronic staffing shortage and requires the flexibility to assign additional staff on days with heavy or unpredictable surgical load. Surplus assignment is not left uncontrolled: any assignment beyond the 20-day monthly ceiling is penalized through the monthly working-day goal constraint (Equation (14)), so overstaffing among permanent nurses is restrained indirectly through the workload-equity goals rather than through a separate coverage penalty. The duty load of the subcontracted pool is restrained instead by the recovery-period goals, which prevent consecutive extended duties.

2. There are 7 subcontracted nurses assigned to night duties. Accordingly, at most 7 nurses can be assigned to this shift, and subcontracted nurses are not assigned to stand-alone day shifts.

For the day shift:

(3) $$\sum_{l=1}^{7}Y_{lj1}=0;\mathrm{all}\;j:\;1,2,\ldots ,28$$

For the night shift:

(4) $$\sum_{l=1}^{7}Y_{lj2}+\sum_{l=1}^{7}Z_{lj2}\leq 7;\mathrm{all}\;j:\;1,2,\ldots ,28$$

3. The seven subcontracted nurses form a single staffing pool. On any given day, a subcontracted nurse may be assigned to either a 16 h duty or a 24 h duty, but not both simultaneously.

(5) $$Y_{lj2}+Z_{lj2}\leq 1;\mathrm{all}\;j:1,2,\ldots ,28\;\mathrm{and}\;l:\;1,\ldots ,7$$

A 24 h duty runs from 08:00 on day j to 08:00 on day j + 1 and therefore spans the day period as well as the night period of day j. The two shift-indexed variables representing that duty must consequently take the same value: a subcontracted nurse contributes to day coverage on day j if and only if that nurse is actually assigned a 24 h duty beginning on day j. Equation (6) enforces this equivalence. Without it, day coverage could be satisfied by a variable that carries no corresponding duty.

(6) $$Z_{lj1}=Z_{lj2};\mathrm{all}\;l:1,\ldots ,7\;\mathrm{and}\;j:1,2,\ldots ,28$$

A permanent nurse may be assigned to at most one shift on any calendar day. Assignment to both the day and the night shift of the same day would amount to a continuous 08:00–24:00 duty, which in this department is reserved for the subcontracted 16 h and 24 h rotations and is not available to permanent staff; it would also cause a single calendar day to be counted twice in the monthly working-day totals. Equation (7) enforces this condition.

(7) $$X_{ij1}+X_{ij2}\leq 1;\mathrm{all}\;i:1,\ldots ,37\;\mathrm{and}\;j:1,2,\ldots ,28$$

#### 2.3.4. Flexible Constraints

A deliberate distinction is drawn between conditions that define feasibility and conditions that represent penalized scheduling goals. Only the coverage requirements (Equations (1) and (2)) and the structural conditions governing the subcontracted staffing pool, together with the one-shift-per-day condition for permanent nurses (Equations (3)–(7)), are enforced as obligatory constraints: a schedule that violates them is not operationally admissible. The weekly night-shift limit, the night-to-day transition condition, and the monthly working-day bounds are operational targets that may, under constrained staffing conditions, need to be violated at a penalty rather than rendering the model infeasible. They are therefore modeled exclusively as goal constraints with deviation variables and are not additionally imposed as obligatory inequalities. This avoids redundancy between feasibility conditions and penalized goals and ensures that every deviation variable appearing in the objective function has an unambiguous interpretation as the extent of a permitted violation. Table 5 summarizes this distinction.

Flexible constraints improve the quality of the schedule within the feasible region defined by the obligatory constraints. Goal constraints 1 to 7 are each expressed as an equality with a target value, together with a pair of non-negative deviation variables, in the form (achieved value) − _Sn_^−^ + S_n_^+^ = target; goal constraints 8 and 9 instead use the binary violation indicators. The objective function penalizes only the deviation corresponding to the undesirable direction. For the weekly night-shift goals, only S_n_^−^ is penalized, since assigning fewer than two night shifts in a week is not undesirable. For the monthly working-day goals, only S_6i_^+^ (falling below the 18-day floor) and S_7i_^−^ (exceeding the 20-day ceiling) are penalized, since the two goals act together: any allocation that leaves both complementary deviations unpenalized lies inside the acceptable 18–20 day band. For the recovery-period goals, the binary indicators S_8lj_ and S_9lj_ are penalized whenever the corresponding rest period is not respected.

The five flexible constraints, three derived from the interview themes and two from the regulatory recovery requirements, are:

- Night duties must not exceed two per nurse in any week.
- On any given day, a nurse assigned to the night shift must not be allocated to the day shift on the following day.
- Each nurse must work between the minimum and the maximum number of days per four-week cycle.
- A subcontracted nurse must have two rest days after a 16 h duty.
- A subcontracted nurse must have three rest days after a 24 h duty.

Penalty cost weights for these five flexible constraints were determined in consultation with the head nurse of the operating room department, on the basis of the scheduling concerns identified in the nurse interviews and the regulatory recovery requirements. The weights applied were: C_1_^−^ = C_2_^−^ = C_3_^−^ = C_4_^−^ = 80 for the four weekly night-shift goals; C_5_^−^ = 60 for the night-to-day transition goal; C_6_^+^ = C_7_^−^ = 100 for the monthly working-day goals; and C_8_ = C_9_ = 500 for the 16 h and 24 h recovery-period goals. The complementary deviations (C_1_^+^–C_4_^+^, C_5_^+^, C_6_^−^, C_7_^+^) are not penalized and are assigned a weight of zero, for the reasons set out above.

The penalty weights were established through expert-based preference elicitation conducted with the head nurse, who holds formal responsibility for preparing the departmental roster and therefore has direct operational knowledge of how frequently each requirement was breached under the manual system and what the consequences were for the department. The scheduling concerns to be weighted were those identified in the thematic analysis of the nurse interviews, together with the regulatory recovery requirements (Table 4); the relative priority attached to them was determined in consultation with the head nurse rather than by the nurses themselves. In that consultation, each constraint was considered against three criteria: its operational impact on daily workflow, whether it derived from a regulatory recovery requirement, and the frequency with which it had been violated under the manual system. Constraints deriving from regulatory recovery requirements were placed in the highest priority class; workload-equity constraints in the second; weekly night-shift limits in the third; and the night-to-day transition constraint in the lowest, since it may be permitted under emergency conditions. These four ordinal priority classes were translated into the cardinal weights 500, 100, 80 and 60, respectively, chosen to preserve the priority ordering in the relative magnitude of the weights while keeping all goals on a single commensurable scale, as summarized in Table 6. The rationale column of Table 6 uses nurse health and fatigue reduction in the restricted senses defined in Section 2.4: they denote scheduling patterns identified in prior literature as impairing recovery, not outcomes measured in this study. Two limitations of this procedure should be stated explicitly: the weights reflect the judgment of a single expert informant rather than an aggregated preference across the nursing staff, and the translation from ordinal priorities to cardinal values necessarily involves judgment. The robustness of the resulting schedule to the specific values chosen was therefore tested in the sensitivity analysis reported in Section 3.4.

The formulation is a weighted (Archimedean) goal programming model rather than a preemptive one. The four priority classes described above are ordinal, but they are combined into the single weighted objective of Equation (17) rather than optimized in sequence, so the ordering they express is not enforced lexicographically: a sufficiently large number of low-weight deviations can outweigh one high-weight deviation. Seven units of the weekly night-shift deviation (7 × 80 = 560), for example, cost more than a single recovery-period violation (500). The weights therefore express the relative cost the department attaches to each type of violation rather than a strict hierarchy among them. A preemptive formulation would enforce the hierarchy exactly, but at the price of admitting no trade-off between classes; it was not adopted because the department’s own practice permits such trade-offs under emergency conditions, for the reasons set out below. The distinction is not operative in the optimized schedule, where every penalized deviation is zero; it would become operative under staffing conditions in which the goals compete.

To ensure operational feasibility and to reflect real-world clinical practice, the conditions in the model are separated into obligatory and flexible constraints (termed hard and soft constraints in the wider rostering literature). The obligatory constraints enforce what cannot be traded away: the daily coverage requirement of the department and the structural conditions governing shift eligibility and the subcontracted staffing pool (Equations (1)–(7)). The recovery periods following extended duties (two rest days after a 16 h duty and three after a 24 h duty) are instead modeled as goal constraints carrying the highest penalty weight in the model (500).

This choice reflects the way shift scheduling is actually governed in Turkish inpatient institutions. Under Article 41 of the Regulation on the Operation of Inpatient Treatment Institutions [42], a member of staff who has worked a night duty is not assigned duty on the following day, and longer rest may be granted where conditions permit; the provision applies to nursing staff as well as to physicians. The two- and three-day windows applied in this department therefore extend a regulatory minimum rather than restate one. Under the same regulation, on-call and shift services are organized and approved administratively at the departmental and institutional levels, taking into account operational dynamics, staff leave and unexpected staffing shortages. The recovery periods are therefore a regulatory and institutional requirement exercised with a defined margin of administrative discretion, rather than an absolute prohibition. Modeling them as high-penalty goal constraints preserves that discretion: it prevents the model from becoming mathematically infeasible during severe understaffing or an emergency surge while ensuring that the recovery periods are respected under all normal operating conditions, as they are in the optimized schedule reported in Section 3.2, where both associated deviations are zero. This is also why the manuscript refers to regulatory rather than strictly legal constraints.

**Flexible 1:** Night duties within any one week should not exceed two per nurse. Under the manual system, individual nurses were frequently assigned more than two night shifts within a single week. Equations (8)–(11) express this requirement as a goal constraint for each of the four 7-day planning blocks, using deviation variables. A weight of 80 is assigned to the excess deviation S_ni_^−^; the complementary deviation, corresponding to a nurse being assigned fewer than two night shifts in a week, is not penalized.

(8) $$\sum_{j=1}^{7}X_{ij2}-S_{1i}^{-}+S_{1i}^{+}=2;\mathrm{all}\;i=1,\ldots ,37$$

(9) $$\sum_{j=8}^{14}X_{ij2}-S_{2i}^{-}+S_{2i}^{+}=2;\mathrm{all}\;i=1,\ldots ,37$$

(10) $$\sum_{j=15}^{21}X_{ij2}-S_{3i}^{-}+S_{3i}^{+}=2;\mathrm{all}\;i=1,\ldots ,37$$

(11) $$\sum_{j=22}^{28}X_{ij2}-S_{4i}^{-}+S_{4i}^{+}=2;\mathrm{all}\;i=1,\ldots ,37$$

**Flexible 2:** On any given day, if a nurse is assigned to the night shift, that nurse should not be assigned to the day shift on the following day. Under the manual system, this transition occurred routinely, and the shift-work literature links this pattern to accelerated fatigue and reduced care quality [8,37]. Equation (12) expresses this requirement as a goal constraint. The excess deviation S_5ij_^−^ carries a weight of 60, the lowest in the model, so that the solver may accept such a transition where it is required to maintain coverage under emergency conditions.

(12) $$X_{i(j+1)1}+X_{ij2}-S_{5ij}^{-}+S_{5ij}^{+}=1;\mathrm{all}\;i=1,2,\ldots ,37;j=1,2,\ldots ,27$$

**Flexible 3:** These constraints specify the minimum and maximum number of working days for each nurse over the four-week scheduling cycle. The departmental target is 19 working days per nurse per cycle, within an acceptable band of 18 to 20 days. Equations (13) and (14) express the lower and upper bounds as goal constraints, with a weight of 100 assigned to S_6i_^+^ (falling below 18 days) and to S_7i_^−^ (exceeding 20 days).

(13) $$\sum_{j=1}^{28}(X_{ij1}+X_{ij2})-S_{6i}^{-}+S_{6i}^{+}=18;\mathrm{all}\;i=1,\ldots ,37$$

(14) $$\sum_{j=1}^{28}(X_{ij1}+X_{ij2})-S_{7i}^{-}+S_{7i}^{+}=20;\mathrm{all}\;i=1,\ldots ,37$$

**Flexible 4:** A 16 h duty requires two rest days afterwards. After a subcontracted nurse works a 16 h duty on day j, that nurse should not be assigned to any further duty (16 h or 24 h) on days j + 1 or j + 2. Because a subcontracted nurse may perform either duty type, the requirement is enforced over the combined duty load. The binary violation indicator S_8lj_ takes the value 1 whenever a 16 h duty on day j coincides with any duty within its rest window and is penalized in the objective function with a weight of 500. This requirement is expressed in Equation (15).

(15) $$S_{8lj}\geq Y_{lj2}+\left(Y_{l\left(j+d\right)2}+Z_{l\left(j+d\right)2}\right)-1;for\;all\;l=1,\ldots ,7;j=1,\ldots ,27;\;d\in \left\{1,2\right\},\;j+d\leq 28$$

**Flexible 5:** Following a 24 h duty on day j, the subcontracted nurse must have three consecutive rest days (j + 1, j + 2, j + 3) before any subsequent duty assignment. The binary violation indicator S_9lj_ takes the value 1 whenever this three-day recovery window is not respected and is penalized in the objective function with a weight of 500. This requirement is expressed in Equation (16).

(16) $$S_{9lj}\geq Z_{lj2}+\left(Y_{l\left(j+d\right)2}+Z_{l\left(j+d\right)2}\right)-1;for\;all\;l=1,\ldots ,7;j=1,\ldots ,27;\;d\in \left\{1,2,3\right\},\;j+d\leq 28$$

Although five flexible constraints are defined conceptually, Flexible Constraint 1 is decomposed into four weekly goal constraints (one for each 7-day planning block) and Flexible Constraint 3 into two bound constraints, so that the model contains nine goal constraints in total (Equations (8)–(16)), indexed n = 1, …, 9.

#### 2.3.5. Goal Function

The goal function is defined not over the decision variables but over the deviation variables of the goal constraints. The aim is to minimize the weighted sum of the penalized deviations. Equation (17) represents the objective function of the scheduling system. As noted above, the weights were determined through structured consultation with the head nurse and reflect the priority ordering set out in Table 6.

(17) $$Min\;Z=80\sum_{i}\left(S_{1i}^{-}+S_{2i}^{-}+S_{3i}^{-}+S_{4i}^{-}\right)+60\sum_{ij}S_{5ij}^{-}+100\sum_{i}\left(S_{6i}^{+}+S_{7i}^{-}\right)+500\sum_{lj}(S_{8lj}+S_{9lj})$$

### 2.4. Operational Definitions

This subsection sets out the operational definition adopted in this study for each key term, so that the components of the model and its outcome are interpreted consistently. The definitions delimit what the study measures and, equally important, what it does not; they are used in the same sense throughout Section 3 and Section 4.

A *goal programming model* is understood here as a multi-objective optimization formulation in which each scheduling requirement is expressed as a goal constraint with an associated target value, with departures from those targets quantified by non-negative deviation variables and minimized in a single weighted objective function. The formulation is of the weighted (Archimedean) type: all goals enter one objective and are traded off against one another through their weights, rather than being optimized in a strict priority sequence. The *components of the model* are the input parameters (Section 2.3.1), the binary decision variables (Section 2.3.2), the obligatory constraints defining feasibility (Equations (1)–(7)), the nine goal constraints with deviation variables (Equations (8)–(16)), and the weighted objective function (Equation (17)). The *model outcome* is a 28-day binary assignment schedule specifying, for each nurse and each day, whether that nurse is assigned to a day shift, a night shift, a 16 h duty, a 24 h duty, or no duty.

Three terms relating to nurse well-being are used in a deliberately restricted sense. *Nurse health* is operationalized as the frequency of scheduling patterns known from prior literature to impair recovery, namely, the weekly night-shift count and the presence of night-to-day transitions [8,9,37]; no clinical or physiological health measure was collected. *Fatigue reduction* is used only as a potential implication inferred from prior literature [8,9,37,40]; no fatigue instrument or scale was administered in this study. *Workload equity is measured by the statistical dispersion of monthly working days* across the 37 permanent nurses, measured by the standard deviation, coefficient of variation, and Gini coefficient; it does not cover shift intensity, case complexity, seniority, or perceived fairness.

The remaining terms describe the scheduling requirements themselves. A requirement is frequently violated if it was breached at least once per week on average across the four-week historical record. *Strong dissatisfaction* denotes a scheduling concern that recurred across multiple interviews and was identified as a distinct theme during the inductive coding process, rather than an isolated individual complaint; because frequencies were not tallied during the thematic analysis, the term denotes a qualitative pattern of recurrence rather than a counted proportion. A regulatory recovery requirement is a post-duty rest provision deriving from Article 41 of the Regulation on the Operation of Inpatient Treatment Institutions [42], as applied in this department through the two- and three-day recovery windows described in Section 2.3.4. The term regulatory is used in preference to legal because the provision is applied administratively, with a defined margin of institutional discretion, as Section 2.3.4 explains. Emergency exceptions allowed refers to the lowest penalty weight in the model (60), carried by the night-to-day transition goal, which permits the solver to accept such a transition where it is required for feasibility under emergency coverage demand.

## 3. Results

### 3.1. Model Implementation and Solver Performance

The goal programming model was implemented in Python 3.11.4 using Google OR-Tools version 9.8.3296 and solved with the CP-SAT solver on a machine with an Intel Core i7-9700K processor, 64 GB of RAM, running Windows 11 Pro. The solver was run with its default settings, including default multi-threading; no parameter was changed. The optimal schedule was generated for a 28-day scheduling cycle covering 37 permanent nurses and 7 subcontracted nurses across day shifts, 16 h night duties, and 24 h night duties. The data used were obtained through interviews with the head nurse and retrieval of scheduling records from the hospital system for the December 2025 period.

**Figure 1:**
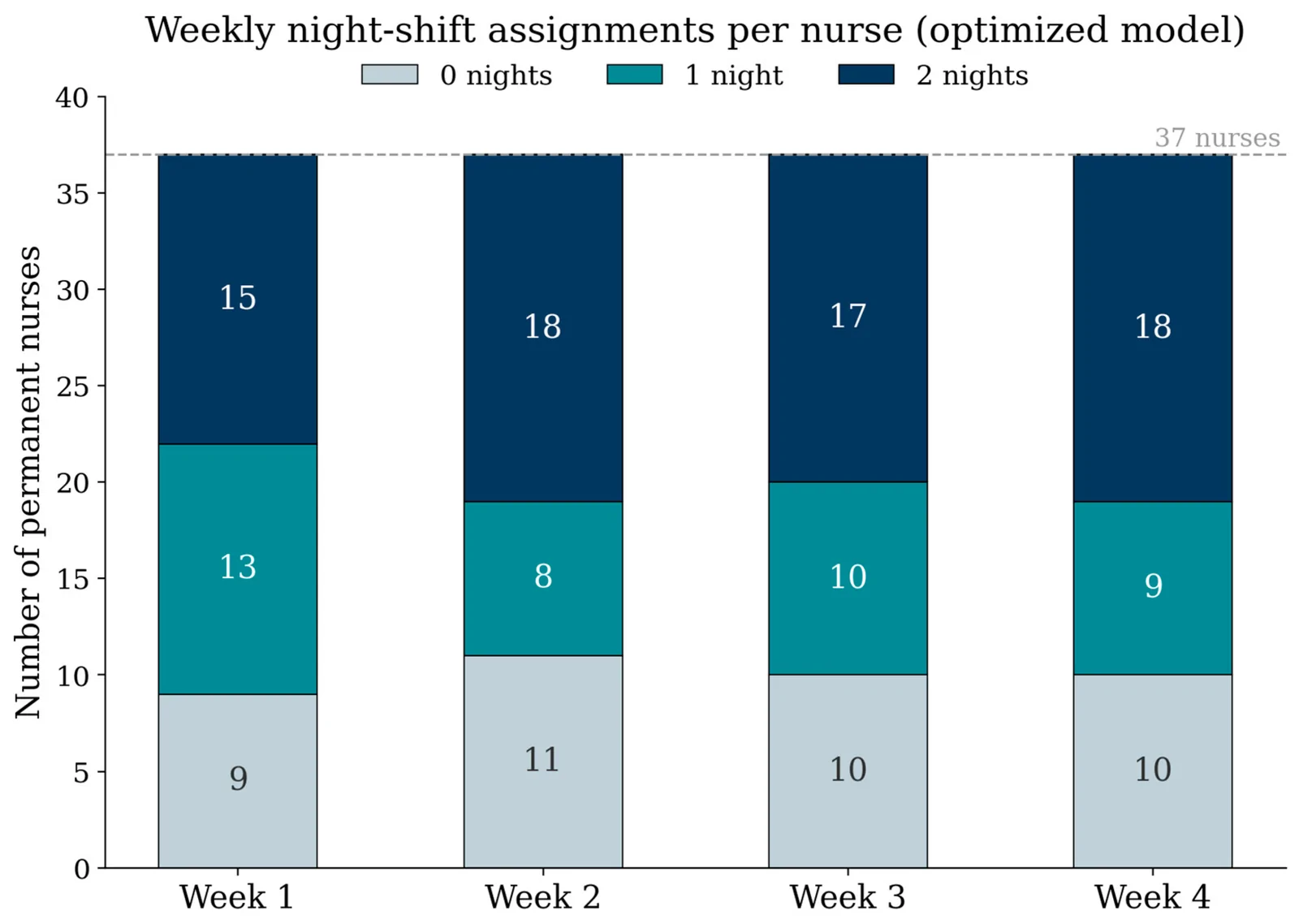
Distribution of weekly night-shift assignments per permanent nurse under the optimized model.

**Figure 2:**
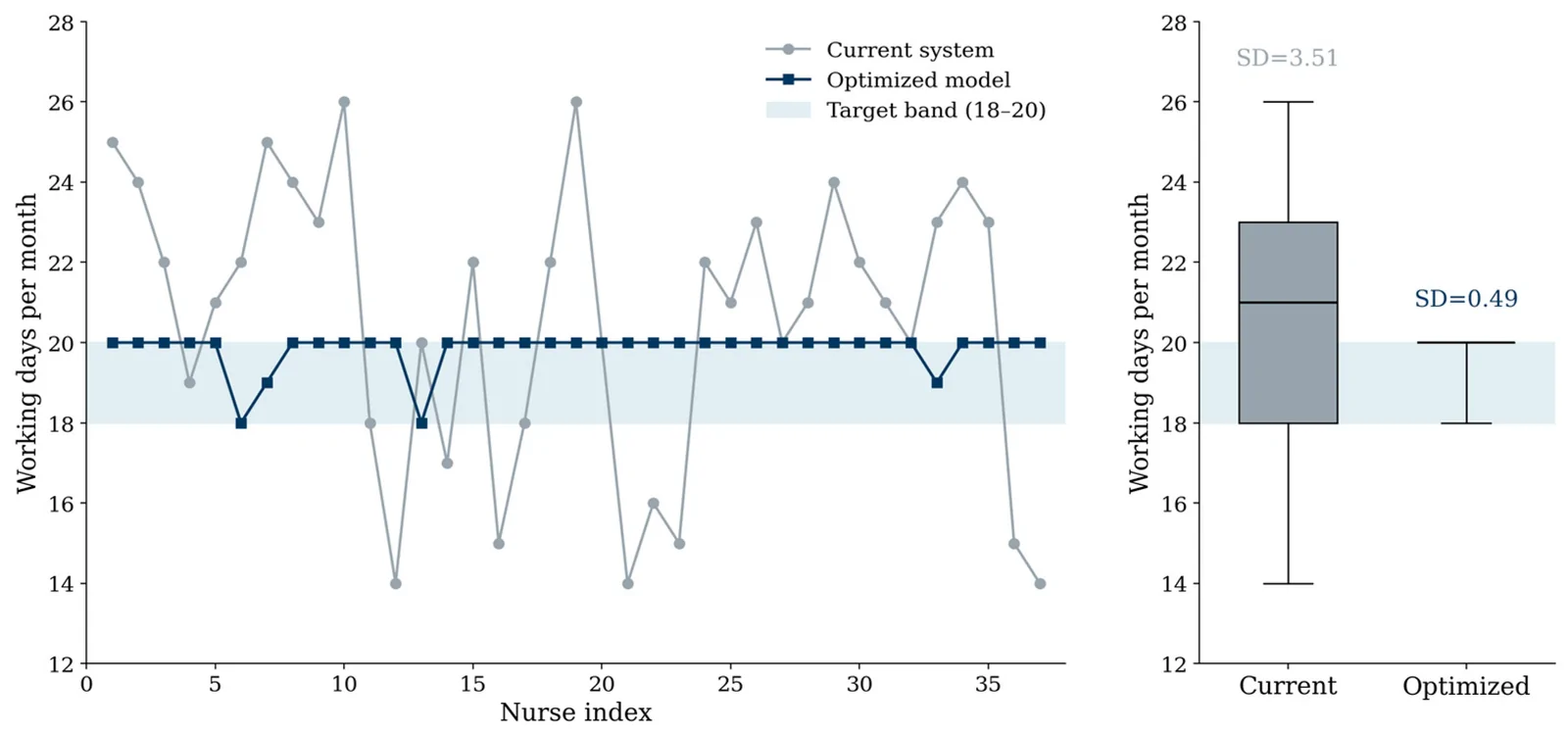
Monthly working days per nurse: current system versus optimized model.

**Figure 3:**
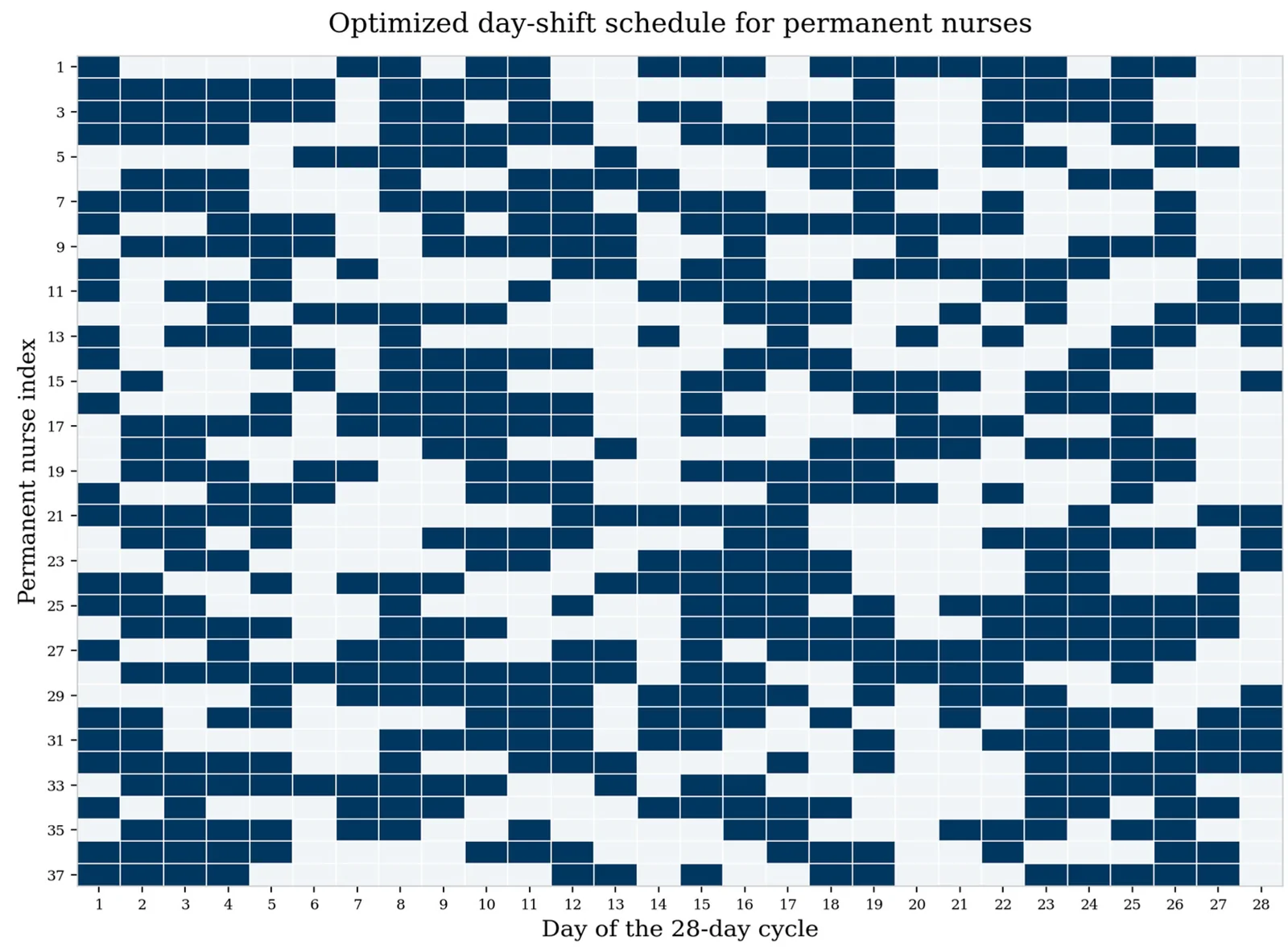
Optimized day-shift schedule for permanent nurses over the 28-day cycle.

**Figure 4:**
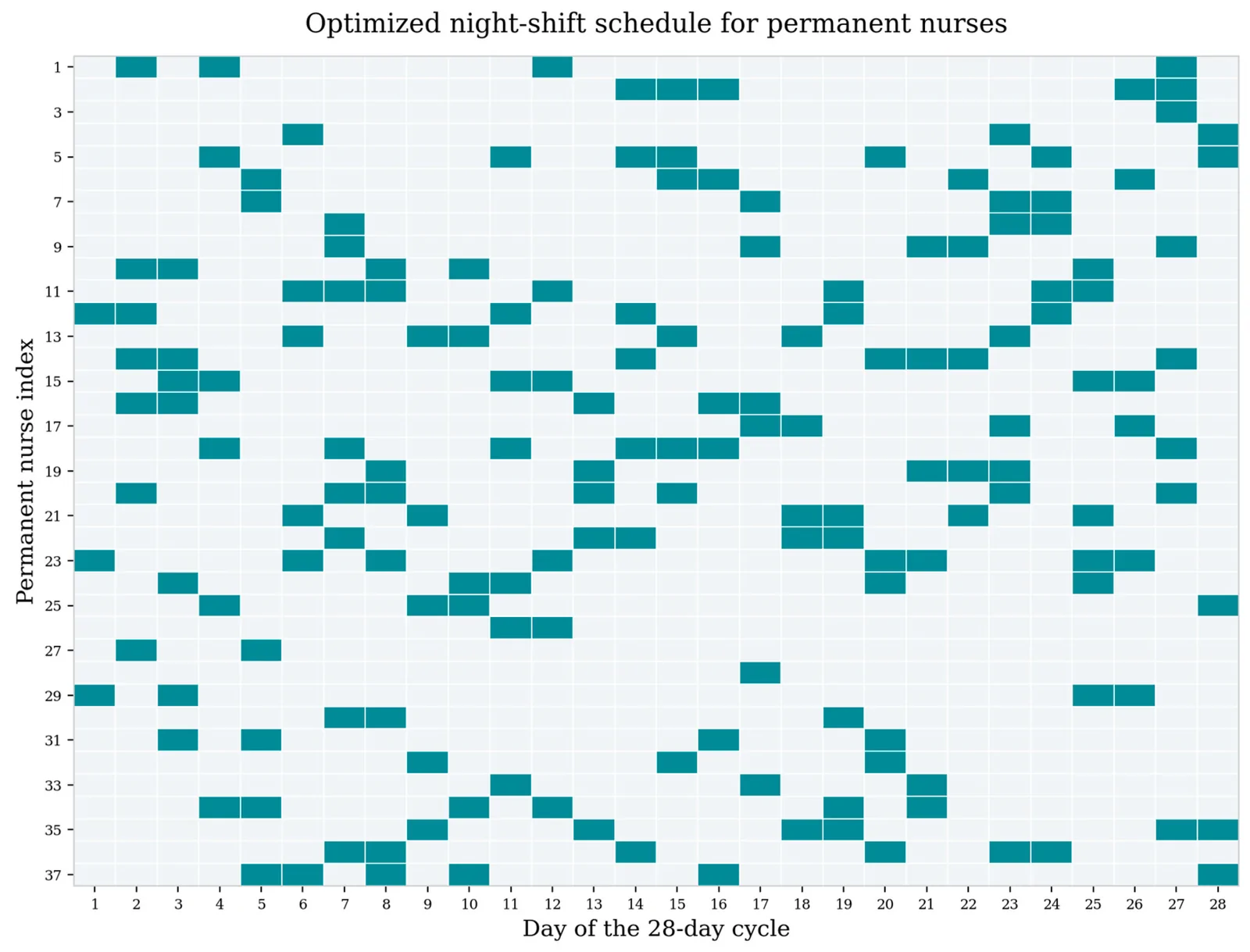
Optimized night-shift schedule for permanent nurses over the 28-day cycle.

**Figure 5:**
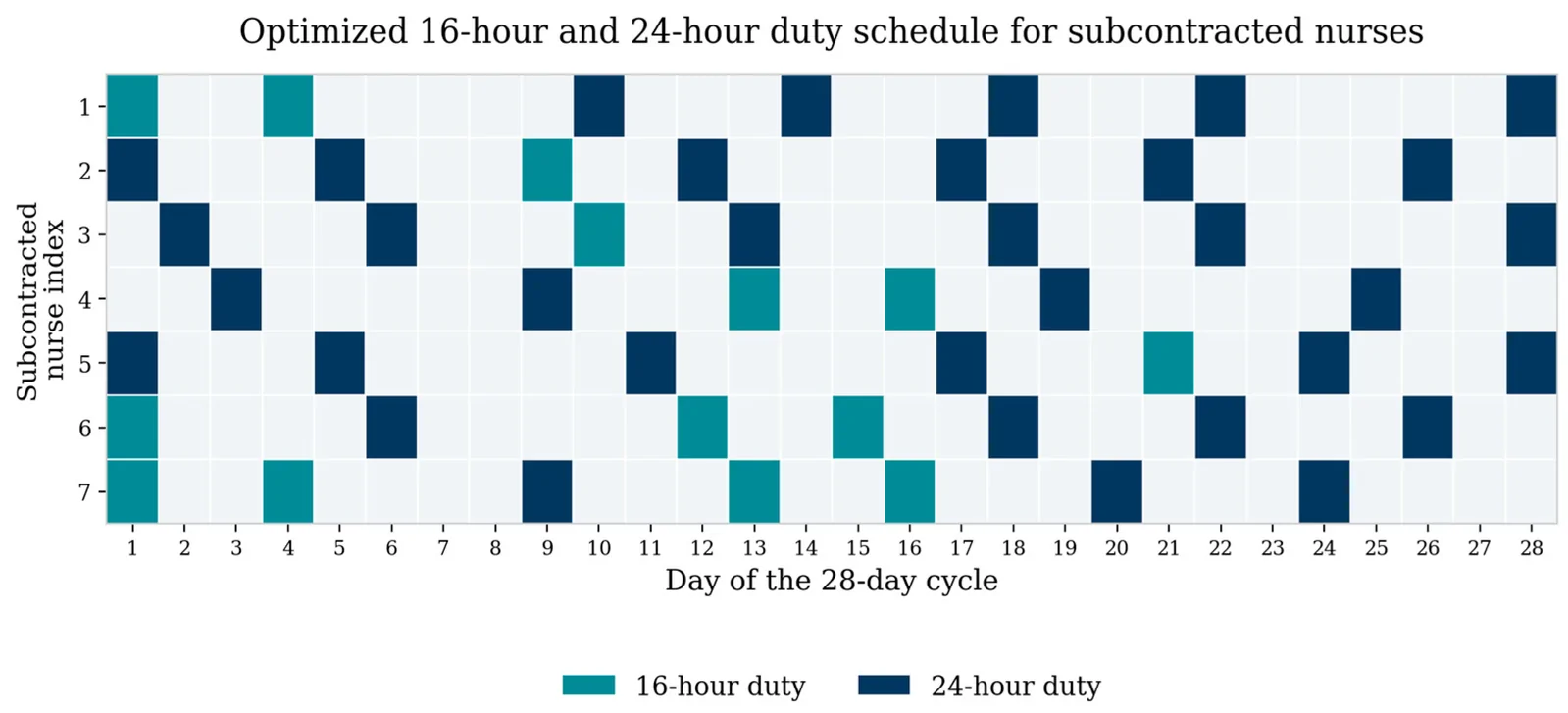
Optimized 16 h and 24 h duty schedule for subcontracted nurses.

The solver returned a proven optimal solution. The final objective function value was 0, with every penalized deviation equal to zero: the weekly night-shift goals (S_1_–S_4_) = 0; the night-to-day transition goal (S_5_) = 0; the monthly working-day goals (S_6_–S_7_) = 0; and the recovery-period goals (S_8_–S_9_) = 0. All five flexible goals were therefore attained simultaneously in the optimized schedule. Proven optimality was reached in 0.7 s on the machine described above. The optimal objective value of zero is attained by more than one schedule, so the particular optimum returned varies between runs. Across five solver seeds the constraint-compliance outcomes were identical in every run (a ceiling of two night shifts per nurse per week, no night-to-day transitions, no recovery-period violations, and monthly working days within the 18–20 day band), while the dispersion statistics varied slightly between optima (mean 19.68–19.92 days, SD 0.34–0.66, CV 0.017–0.034, Gini 0.004–0.014). The values reported below are those of the run whose schedule is presented in Figure 1, Figure 2, Figure 3, Figure 4 and Figure 5. The reported runtime refers to a single optimization run on this instance; it establishes that this particular instance is solved to proven optimality in under a second on a desktop machine and does not establish scalability to larger instances.

### 3.2. Compliance with the Flexible Constraints

With respect to the first flexible constraint, the optimized model enforced the weekly night-shift limit of two for every permanent nurse across all four planning blocks. Figure 1 shows the distribution of weekly night-shift assignments under the optimized model: in each week, every nurse is assigned zero, one, or two night shifts, with none exceeding the two-shift limit. This differs from the manual system, in which night-shift assignments were unevenly distributed and individual nurses were frequently assigned more than two night shifts per week. The distribution shown in Figure 1 confirms that the model satisfies the per-nurse limit individually rather than merely on average. Because the limit is defined over the four fixed 7-day blocks, up to three consecutive night shifts can still occur where a run straddles a block boundary; this affects five nurses in the optimized schedule. A rolling seven-day window would remove this, at the cost of a tighter and possibly infeasible formulation.

With respect to the second flexible constraint, direct night-to-day transitions were eliminated in the optimized schedule. Under the manual schedule, approximately three nurse assignments per week placed a nurse on a day shift on the day immediately following a night shift; the figure is the weekly average across the four-week record, corresponding to approximately twelve such assignments over the 28-day period. The optimized model prevents a day-shift assignment on the day following a night shift for permanent nurses, and no such transition occurs in the resulting schedule.

With respect to the third flexible constraint, working days per month were standardized to a range of 18–20 days for each nurse individually, with a target of 19 days. In the current system, monthly working days ranged irregularly between 14 and 26 days across nurses. Figure 2 compares monthly working days per nurse under the historical manual schedule and under the optimized model.

Although the departmental target is 19 working days, the optimized schedule places 33 of the 37 nurses at 20 days and the remaining four at 18 or 19, giving a mean of 19.84. This follows from the structure of the goal constraints rather than from a departure from the target: deviations are penalized only below the 18-day floor and above the 20-day ceiling, so any allocation inside the band is unpenalized and the solver may settle anywhere within it. Where it settles is set by how much coverage the subcontracted pool absorbs, which the recovery-period goals limit. A mean of exactly 19 is in any case unattainable here: the cycle requires 816 shift assignments in total, of which the seven subcontracted nurses can supply at most 98 under the recovery windows, leaving at least 718 for the 37 permanent nurses and hence a mean of at least 19.4.

With respect to the fourth and fifth flexible constraints, all identified rest-period violations for subcontracted nurses were eliminated in the optimized schedule. Under the historical schedule, at least four assignments per week following a 16 h duty and at least three per week following a 24 h duty fell within the mandated recovery window, giving at least seven violating assignments per week and at least twenty-eight over the four-week period. The unit throughout is violating assignments rather than distinct nurses. In the optimized schedule, every 16 h duty is followed by two rest days and every 24 h duty by three, before any further duty assignment. Figure 3 and Figure 4 present the optimized day-shift and night-shift schedules for permanent nurses, and Figure 5 presents the 16 h and 24 h duty schedule for subcontracted nurses.

### 3.3. Comparison with the Manual Schedule

To evaluate the optimized schedule beyond simple before-and-after comparisons, several standard quantitative indicators of workload equity and constraint compliance were computed and compared against the current manual schedule (Table 7). Workload equity was assessed using the standard deviation (SD), coefficient of variation (CV), and Gini coefficient of monthly working days across the 37 permanent nurses, where lower values indicate a more equal distribution.

Under the current manual system, monthly working days ranged from 14 to 26 (mean = 20.57, SD = 3.51, CV = 0.171, Gini = 0.096), reflecting a wide dispersion in the number of scheduled working days across nurses. The optimized model reduced this dispersion markedly, standardizing working days to an 18–20 range (mean = 19.84, SD = 0.49, CV = 0.025, Gini = 0.007). The reduction in standard deviation from 3.51 to 0.49 represents an 86% decrease in the dispersion of monthly working days, and the Gini coefficient of 0.007 indicates a substantially more equal distribution of scheduled working days across the workforce.

These indicators describe the statistical equality of the number of scheduled working days only. They do not capture differences in shift duration, night-shift burden, case complexity, seniority, or individual preference and should therefore not be read as a measure of fairness in a broader organizational or clinical sense.

The weekly night-shift ceiling was respected throughout: under the optimized model, no nurse exceeded two night shifts in any weekly block; each nurse worked zero, one, or two nights per week (Figure 1). The ceiling constrains the weekly count and not the cycle total, so the number of night shifts a nurse works across the 28 days still varies, from one to eight. Night-shift burden was therefore not equalized, and the equity indicators above, which describe scheduled working days, should not be read as covering it. All constraint-violation indicators, covering rest-period violations for both 16 h and 24 h duties and night-to-day transitions, were reduced to zero in the optimized solution.

### 3.4. Sensitivity to the Penalty Weights

Because the penalty weights were determined through expert-based preference elicitation and therefore carry a degree of subjectivity, a sensitivity analysis was conducted to assess the robustness of the optimized schedule to weight variation. The four groups of penalty weights (the weekly night-shift limits C_1_–C_4_, the night-to-day transition penalty C_5_, the monthly workload-balance penalties C_6_–C_7_, and the recovery-period penalties C_8_–C_9_) were each perturbed by ±20% and ±50% relative to their baseline values, and the model was re-solved under each of the sixteen resulting scenarios. Across all scenarios, the model continued to reach proven optimality with an objective value of zero, and every compliance outcome was identical to the baseline result: full recovery-period compliance, individual weekly night-shift limits, elimination of night-to-day transitions, and monthly working days inside the 18–20 day band. The particular optimum returned varied between scenarios, as it does between solver seeds, so the dispersion statistics differ slightly across the sixteen runs.

This invariance indicates that, under the specific staffing structure and demand profile of the studied department, the five flexible goals are simultaneously satisfiable and do not compete with one another. It should not be generalized as an absence of trade-offs in the model itself. Rather, it suggests that the current staffing level provides sufficient slack for all goals to be met concurrently and that the cardinal values of the penalty weights become operationally decisive only under more constrained conditions (reduced staffing, elevated surgical demand, or extended planning horizons) in which the goals would come into direct competition. In practical terms, the weighting structure functions here as a safeguard for constrained scenarios rather than as an active discriminator in the present configuration. The invariance concerns the relative values of the weights, not whether the goals bind at all: the ablation analysis in Section 3.5 shows that removing the goal constraints altogether produces a schedule with 30 night-to-day transitions and 189 recovery-period violations. Because the invariance is a property of this instance rather than of the model, an institution adopting this formulation should repeat the sensitivity analysis on its own staffing and demand data rather than rely on the result reported here.

### 3.5. Baseline and Ablation Analysis

To isolate the incremental contribution of the flexible constraints, the model was solved under three specifications: (i) a baseline containing only the obligatory constraints (Equations (1)–(7)); (ii) an intermediate specification adding the three permanent nurse goal constraints (Flexible 1–3, Equations (8)–(14)); and (iii) the full model, which additionally includes the two recovery-period goals for subcontracted nurses (Flexible 4–5, Equations (15) and (16)). Table 8 reports the resulting schedules against the same indicators used for the comparison with the historical schedule.

Because specifications (i) and (ii) leave the omitted requirements unpenalized, the solver is indifferent among all schedules that are optimal for that specification; the values reported for these columns are those returned under identical solver settings. Because the recovery-period indicators carry no penalty in specifications (i) and (ii), the violation counts in those columns are recomputed directly from the returned schedules rather than read from the indicator variables. The obligatory constraints alone place no restriction on the distribution of night shifts, on night-to-day transitions, or on recovery periods: the solver returns a schedule in which every nurse is assigned on all 28 days, with up to seven night shifts in a single week, 30 night-to-day transitions and 189 recovery-period violations. No 16 h duty is assigned under this specification, so every recovery-period violation it contains follows a 24 h duty. The dispersion statistics of that schedule are trivially zero, because every nurse works every day; this illustrates concretely why equality of scheduled working days is not by itself a measure of a good or fair roster. Additionally, adding Flexible 1–3 resolves the permanent nurse objectives but leaves the subcontracted recovery periods unprotected, which is precisely the gap that Flexible 4–5 closes. The flexible constraints therefore do not enlarge the set of achievable schedules, since a compliant schedule is feasible under the obligatory constraints alone; they are what cause the solver to return one.

## 4. Discussion

### 4.1. Principal Findings

This study developed and applied a goal programming model to optimize nurse shift scheduling in an operating room department, integrating five flexible constraints derived from nurse preferences and institutional requirements. The optimized model generated a schedule with improved compliance with the predefined scheduling objectives across all five constraint dimensions, compared with the historical manual schedule.

### 4.2. Comparison with Prior Work and Scheduling Trade-Offs

The enforcement of a per-nurse ceiling of two night shifts per week, a limit breached for individual nurses under the manual system, is consistent with Wong et al. [29], who address night-shift accumulation through soft constraints in an emergency department setting. The dual-tier structure of the roster is comparable to the reserve-pool arrangement of Saemi [23], in which a distinction between the regular establishment and a reserve pool gives the scheduler a second source of night coverage. Direct night-to-day shift transitions were eliminated completely.

While prior literature associates night-to-day transitions with cognitive fatigue and reduced care quality [8,37], the present study did not measure fatigue, cognitive performance, care quality, or error rates. The elimination of these transitions is therefore reported as a scheduling outcome with potential well-being implications rather than as a demonstrated reduction in fatigue. This change nevertheless carries organizational implications that warrant consideration. Preventing a day-shift assignment on the day after a night shift reduces the pool of nurses available for day-shift assignment on any given day, which in a chronically understaffed department effectively increases the coverage burden on the remaining staff. In the present case, part of the night coverage was carried by the subcontracted pool, but the recovery windows limit how densely that pool can be used: each subcontracted nurse can work at most seven duties in a 28-day cycle, and the optimized schedule uses 48 duties in total, leaving the remaining night coverage to the permanent establishment. In departments without such a pool, the same constraint could increase reliance on overtime or temporary staffing, with associated cost implications. This illustrates a fundamental trade-off in nurse scheduling: improvements in staff well-being and rest adequacy may reduce short-term staffing flexibility, and the net operational effect depends on the availability of supplementary staffing capacity. The proposed model does not eliminate this trade-off but renders it explicit and manageable through the penalty-weight structure, allowing managers to calibrate the balance between well-being and flexibility according to institutional priorities. This weighted-deviation approach is consistent with recent multicriteria scheduling formulations that employ penalty structures to balance fairness and rest-related objectives [43], though the present study extends such formulations to the dual-tier, heterogeneous-shift operating room context.

The trade-off also has an economic dimension. The optimized schedule changes how night work is divided between the permanent establishment and the subcontracted pool. The manuscript does not quantify that change against the manual roster, because the subcontracted workload under the manual schedule was not recorded at that level of detail. Subcontracted capacity is procured under an external service contract and is not costless, so any change in the division of duties carries financial consequences for the department that the present objective function does not represent. No costing data were available for this study, and the model optimizes scheduling outcomes alone. Any institution adopting this formulation should therefore evaluate the staffing cost consequences of the resulting roster alongside its equity and compliance benefits, and an explicit cost term is a natural extension of the objective function.

Standardizing monthly working days to 18–20 days addresses what Schoenfelder et al. [19] identified as a central challenge: balancing workload equity across staff while meeting coverage requirements. In the current system, the range of 14–26 working days per month creates simultaneous conditions of underutilization and overwork, a pattern documented in other studies of manual scheduling [41,44,45]. The proposed model reduces this variability substantially, from a standard deviation of 3.51 days to 0.49.

A similar tension exists between workload equity and operational efficiency. Standardizing monthly working days to a narrow 18–20-day band improves fairness but reduces the scheduler’s freedom to concentrate assignments on a smaller group of nurses during peak demand. In settings with highly variable surgical volume, an overly rigid equity constraint could in principle conflict with coverage requirements. The goal programming formulation accommodates this tension by treating equity as a weighted flexible constraint rather than an obligatory requirement, so that coverage obligations, encoded as obligatory constraints, are never violated even when full equity cannot be achieved. This design choice distinguishes the present approach from hard-constraint formulations that may become infeasible under demand variability, a trade-off also noted by Jafri et al. [30] in moving between goal programming and binary integer formulations of the nurse rostering problem.

### 4.3. Methodological Contribution and Practical Implications

The methodological differences that underlie the present model’s contribution go beyond consistency with prior work. Mohammadian et al. [24] and Ariyani et al. [25] applied goal programming in settings where all nurses share a homogeneous shift structure; their models therefore do not require the differentiated recovery-period constraints (two days after 16 h duties, three days after 24 h duties) that are essential in the present operating room context. Agyei et al. [26] incorporated legal scheduling regulations but treated all staff as a single category, without the dual-tier permanent–subcontracted distinction that governs night coverage in our setting. Gür et al. [35] combined constraint and goal programming in a surgical setting but addressed the assignment of surgical teams and operations rather than the recovery entitlements of a dual-tier nursing pool. In the present case, the simultaneous enforcement of heterogeneous shift recovery and dual-tier staffing eliminated rest-period violations affecting at least seven violating assignments per week under the manual schedule. No prior formulation was run on these data, so this measures the model against the manual roster rather than against the cited studies. The cited formulations, as published, do not model a dual-tier staffing pool, and only Agyei et al. [26] include heterogeneous duty lengths, without the differentiated recovery entitlements attached to them here. A component-by-component comparison is presented in Table 9. The contribution is therefore an extension of goal programming to a constraint structure that mirrors the operational reality of operating room scheduling.

The boundaries of this contribution should be stated clearly. The incorporation of nurse preferences and soft constraints is, in itself, well established in the nurse rostering literature and does not constitute a novel methodological device. Rather, the contribution of this study lies in the specific adaptation of goal programming to operating room environments characterized by heterogeneous 16 h and 24 h shift structures, differentiated recovery requirements, and a dual-tier staffing model, a combination that has received limited attention in prior work. Framing the contribution at this level avoids overstating the novelty of the underlying technique while accurately positioning this study’s actual advancement.

From a practical standpoint, the proposed model provides nurse managers with a potentially adaptable decision-support tool that requires validation in other settings. The Python implementation can be adapted to other hospital departments by modifying the nurse index range, shift structure parameters, and penalty weights; for departments with different staffing compositions or regulatory frameworks, the flexible constraint structure allows straightforward modification without altering the core model architecture. However, the evidence base is at present limited to a single department over one 28-day scheduling period, and adaptability of the formulation should not be read as demonstrated transferability of the results.

### 4.4. Limitations

Several limitations should be acknowledged. First, the model was developed and validated within a single operating room department of one hospital, which limits the generalizability to settings with different staffing structures or shift regulations; institutions operating under different labor regulations may require substantial reformulation of the constraint set. Second, the scheduling optimization covers a fixed four-week period and is solved against a representative daily staffing requirement rather than the day-by-day requirement actually observed, so it does not account for dynamic variability in patient demand, unplanned nurse absences, or emergency staffing requirements; incorporating stochastic elements, as in the stochastic programming formulation of Bagheri et al. [28] and the dynamic-demand adjustment of Aydas et al. [33], would strengthen real-world applicability. Third, penalty weights were determined through expert-based preference elicitation with the head nurse alone. Although she holds direct operational responsibility for the roster, the weights therefore represent the judgment of a single expert informant rather than an aggregated preference across the nursing staff, which introduces a degree of subjectivity; the sensitivity analysis showed the outcome to be insensitive to the weights only because the five goals proved simultaneously satisfiable in this department, as Section 3.4 sets out, so it does not establish robustness in general; validation across multiple institutions and the application of a formal multicriteria weighting procedure drawing on the whole staff group would strengthen the weighting approach. Fourth, this study focuses on scheduling equity and nurse preferences without addressing economic dimensions such as staffing cost, overtime expenditure, or the financial implications of how duties are divided between permanent and subcontracted staff; no economic evaluation was performed. Fifth, the model was not prospectively deployed; its performance was evaluated by comparing the optimized schedule against retrieved historical records rather than through live implementation, so its real-world sustainability remains untested. Sixth, the scalability of the model has not been evaluated; it remains unclear whether solver performance and solution quality would be maintained in larger hospitals with more nurses, multiple departments, or longer planning horizons. Seventh, the qualitative component was coded by a single researcher without independent verification, and the number of participants raising each theme was not recorded; this may introduce interpretive bias in the translation of interview themes into goal constraints and prevents the relative weight of each concern from being quantified. Eighth, the seven subcontracted nurses were not interviewed, so the preference component of the study covers 37 of the 44 nurses in the rotation, and the two recovery-period constraints rest on regulatory guidance and the head nurse’s account rather than on the preferences of the staff they govern. Finally, the model constrains the weekly night-shift count but not the cycle total or the spacing of nights across a block boundary: night shifts per nurse still range from one to eight over the 28 days, and three consecutive nights occur for five nurses, so night-shift burden is bounded rather than equalized.

Of these limitations, the absence of prospective implementation and the absence of economic evaluation are the most consequential for interpreting the findings. This study demonstrates that an optimized schedule satisfying the stated objectives can be generated; it does not establish that nurses would accept that schedule, that managers could implement it, that coverage would remain adequate in day-to-day practice, or that the division of duties it entails would be financially sustainable.

## 5. Conclusions

This study developed a goal programming model for nurse shift scheduling in an operating room department and solved it to proven optimality using the OR-Tools CP-SAT solver for a 28-day cycle covering 37 permanent and 7 subcontracted nurses. The optimized schedule eliminated direct night-to-day transitions, limited weekly night shifts to a maximum of two per nurse in every planning block, standardized monthly working days to 18–20 days, and eliminated all identified rest-period violations for subcontracted nurses in the optimized schedule.

The conceptual contribution of this work lies in the simultaneous integration of human, operational, and recovery-related constraints within a single optimization framework specifically adapted to operating room scheduling, a setting characterized by heterogeneous 16 h and 24 h shift structures and a dual-tier staffing model. This study advances the application of goal programming and flexible constraints to a constraint structure that mirrors the operational reality of surgical environments, a combination that has received limited attention in prior work.

The proposed model shows potential as a decision-support framework that could be adapted and subsequently validated in other operating room settings. Future work should address the present limitations in order of immediacy. First, prospective implementation with evaluation of nurse acceptance, coverage adequacy, and staff satisfaction is required to move from mathematical feasibility to demonstrated operational effectiveness. Second, an economic evaluation incorporating staffing cost, overtime expenditure, and the financial implications of how duties are divided between permanent and subcontracted staff should be integrated into the objective function. Third, validation across multiple hospitals and departments is needed to assess generalizability and scalability. Fourth, testing under variable and stochastic surgical demand [28,33] would establish robustness, and integration with hospital information systems would allow real-time capture of individual preferences. Only once these have been addressed will comparison with metaheuristic, artificial intelligence, or machine learning approaches [46] become meaningful.

## Figures and Tables

**Table 1:** Comparison of the present study with prior goal programming approaches to nurse scheduling.

Study	Nurse Preferences	Heterogeneous Shifts (16 h/24 h)	Recovery (16 h/24 h)	Dual-Tier Staffing
Mohammadian et al. [24]	✓	✗	✗	✗
Ariyani et al. [25]	✓	✗	✗	✗
Agyei et al. [26]	✓	✓	partial	✗
Rerkjirattikal et al. [38]	✓	✗	✗	✗
Torres-Ramos et al. [27]	✓	✗	✗	✗
Gür et al. [35]	✗	✗	✗	✗
This study	✓	✓	✓	✓

✓ = the component is addressed in the study; ✗ = the component is not addressed.

**Table 2:** Shift types, working hours, duration, eligible staff category and minimum recovery period.

Shift Type	Working Hours	Duration	Staff Category	Minimum Recovery Period
Day shift	08:00–16:00	8 h	Permanent nurses	None
Night shift	16:00–24:00	8 h	Permanent nurses	No day shift on the following day (goal constraint)
16 h duty	16:00–08:00 (+1 day)	16 h	Subcontracted nurses	2 days
24 h duty	08:00–08:00 (+1 day)	24 h	Subcontracted nurses	3 days

**Table 3:** Characteristics of the interview participants, compiled from departmental staffing records provided by the head nurse. SD = standard deviation; n/a = not applicable.

Characteristic	Permanent Nurses (*n* = 37)	Head Nurse (*n* = 1)
Age, mean (SD), years	37.32 (6.19)	40
Age, median (min–max), years	36 (24–52)	n/a
Gender, *n* (%): female/male	32 (86.5)/5 (13.5)	Female
Total nursing experience, mean (SD), years	15.27 (6.39)	18
Operating room experience, mean (SD), years	13.57 (6.07)	15

**Table 4:** Mapping of interview themes and regulatory requirements onto the goal constraints of the model.

Theme/Requirement	Source	Corresponding Goal Constraint	Equations
Excessive accumulation of night shifts within a week	Recurring interview theme (literature-informed guide)	Flexible 1: weekly night duties ≤ 2	(8)–(11)
Assignment to a day shift immediately after night duty	Recurring interview theme; Regulation [42], Article 41	Flexible 2: no night-to-day transition	(12)
Inequitable distribution of monthly working days	Recurring interview theme (literature-informed guide)	Flexible 3: monthly working days 18–20	(13)–(14)
Inadequate rest after a 16 h duty	Regulation [42], Article 41; departmental practice	Flexible 4: two rest days after a 16 h duty	(15)
Inadequate rest after a 24 h duty	Regulation [42], Article 41; departmental practice	Flexible 5: three rest days after a 24 h duty	(16)

**Table 5:** Conditions defining feasibility (obligatory constraints) and conditions representing penalized scheduling goals. n/a = not applicable: obligatory constraints carry no penalty weight.

Requirement	Equation(s)	Role in the Model	Penalty Weight
Day-shift coverage	(1)	Obligatory constraint (feasibility)	n/a
Night-shift coverage	(2)	Obligatory constraint (feasibility)	n/a
Subcontracted nurses not assigned to stand-alone day shifts	(3)	Obligatory constraint (feasibility)	n/a
Subcontracted pool size (≤7 per night)	(4)	Obligatory constraint (feasibility)	n/a
One duty type per subcontracted nurse per day	(5)	Obligatory constraint (feasibility)	n/a
24 h duty spans the day and night periods of the same day	(6)	Obligatory constraint (feasibility)	n/a
One shift per permanent nurse per day	(7)	Obligatory constraint (feasibility)	n/a
Weekly night duties ≤ 2 per permanent nurse	(8)–(11)	Goal constraint (penalized)	80
No night-to-day transition	(12)	Goal constraint (penalized)	60
Monthly working days ≥ 18	(13)	Goal constraint (penalized)	100
Monthly working days ≤ 20	(14)	Goal constraint (penalized)	100
Two rest days after a 16 h duty	(15)	Goal constraint (penalized)	500
Three rest days after a 24 h duty	(16)	Goal constraint (penalized)	500

**Table 6:** Goal constraint weights and rationale.

Flexible Constraint	Weight	Rationale
Weekly night shift limit (≤2/week)	80	Nurse health; frequently violated
Night-to-day transition prohibition	60	Fatigue reduction; emergency exceptions allowed
Monthly working days (18–20)	100	Workload equity; strong dissatisfaction
16 h shift: rest period	500	Regulatory recovery requirement [42]
24 h shift: rest period	500	Regulatory recovery requirement [42]

**Table 7:** Comparison of performance metrics between the current system and the optimized model.

Performance Indicator	Current System	Optimized Model
Monthly working days (mean)	20.57	19.84
Monthly working days (SD)	3.51	0.49
Monthly working days (range)	14–26	18–20
Working-day CV	0.171	0.025
Working-day Gini coefficient	0.096	0.007
Weekly night shifts per nurse	exceeded two for individual nurses	≤2
Rest-period violations (16 h/24 h)	≥4/≥3 assignments per week	0/0
Night-to-day transitions	approx. 3 assignments per week	0

**Table 8:** Baseline and ablation analysis: scheduling outcomes under three model specifications, solved with the solver’s default settings.

Indicator	(i) Obligatory Constraints Only	(ii) Obligatory Constraints + Flexible 1–3	(iii) Full Model
Monthly working days, mean	28.00	18.05	19.84
Monthly working days, SD	0.00	0.32	0.49
Monthly working days, range	28–28	18–20	18–20
Working-day coefficient of variation	0.000	0.018	0.025
Working-day Gini coefficient	0.000	0.003	0.007
Maximum weekly night shifts per nurse	7	2	2
Night-to-day transitions (28 days)	30	0	0
Rest-period violations, 16 h/24 h	0/189	0/189	0/0
Total weighted penalty of the full objective	157,900	94,500	0
Solve time (s)	0.1	0.3	0.7

**Table 9:** Component-by-component comparison of the present model with previous goal programming studies. Column headings give the first author and reference number of each study.

Component	Mohammadian [24]	Ariyani [25]	Agyei [26]	Rerkjirattikal [38]	Gür [35]	This Study
Nurse-health proxy	Night-shift preference	Preference score	Legal rest rules	Individual preference	Not modeled	Weekly night limit + night-to-day goal
Workload-equity metric	Not reported	Not reported	Not reported	Preference satisfaction rate	Operating room utilization	SD, CV, Gini coefficient
Regulatory recovery requirement	Not modeled	Not modeled	Obligatory constraint	Not modeled	Not modeled	High-penalty goal constraint
Heterogeneous shift lengths	No	No	Yes	No	No	Yes (16 h/24 h)
Dual-tier staffing	No	No	No	No	No	Yes
Emergency exception mechanism	No	No	No	No	No	Yes (low-weight goal)
Baseline violation frequency reported	No	No	No	No	No	Yes (28-day historical record)
Solver/optimality status	LINGO	LINGO	LINGO	Not stated	Constraint + goal programming	CP-SAT, proven optimal
Model outcome	Emergency department roster	Ward roster	Ward roster	Ward roster	Surgical team and operation schedule	Operating room nurse roster, 28-day, dual-tier

## Data Availability

Data available on request due to restrictions. The data presented in this study are not publicly available due to institutional confidentiality agreements and privacy considerations related to hospital personnel information.

## Appendix A

**Informed Consent Form For Research Participation**
**Study Title**
A Goal Programming Model for Nurse Shift Scheduling Incorporating Flexible Constraints: A Case Study in an Operating Room Department
**Responsible Researcher**
Name/Title: ____________________________________________________
Institution: ____________________________________________________
E-mail: ____________________________________________________
**1. Purpose Of The Study**
This study aims to develop and implement a mathematical optimization model for nurse shift scheduling in an operating room department. Data regarding working hours, shift patterns, and duty schedules of nursing services are used to formulate flexible constraints that reflect nurse preferences. The findings are intended to contribute to equitable workload distribution, improved working conditions, and enhanced healthcare service efficiency.
**2. Nature Of Participation**
Your participation in this study involves the following:
- Sharing anonymized information related to shift scheduling records and working hour patterns within your department.
- Participating in a semi-structured interview regarding scheduling preferences, workload perceptions, and working conditions (approximately 15–30 min).
- The data collected do not include any personal health information, patient data, or sensitive personal identifiers.
**3. Voluntary Participation And Right To Withdraw**
Participation in this study is entirely voluntary. You have the right to:
- Decline to participate without providing any reason and without facing any negative consequences.
- Withdraw your participation at any time during the study, without penalty or loss of any benefits to which you are otherwise entitled.
- Request the removal of your data from the study at any point prior to the finalization of the dataset.
**4. Confidentiality And Data Protection**
The data collected in this study will be processed in accordance with the following principles:
- All data are of a collective and anonymous nature and do not contain any information that could reveal the identity of any individual participant.
- Data will be used solely for scientific research purposes and will not be shared with third parties.
- Results will be reported in aggregate form only; no individual responses will be identifiable in any publication.
- All records will be stored securely and will be accessible only to the research team.
**5. Participant Declaration**
By signing below, I confirm that:
- I have read and understood the information provided above regarding the nature and purpose of this study.
- I have had the opportunity to ask questions and have received satisfactory answers.
- I understand that my participation is voluntary and that I may withdraw at any time without consequence.
- I consent to the use of the anonymized scheduling data and/or interview responses for the purposes described in this form.

Participant Name Surname: ___________________________________________________ Title/Role: ___________________________________________________ Signature: ___________________________________________________ Date: ___________________________________________________	Researcher Name Surname: ___________________________________________________ Title/Role: ___________________________________________________ Signature: ___________________________________________________ Date: ___________________________________________________
This form has been prepared in two copies. One copy is retained by the participant and one by the researcher.	
For questions or concerns regarding this study, please contact the responsible researcher listed above.	
